# Functions of the Nonsense-Mediated mRNA Decay Pathway in *Drosophila* Development

**DOI:** 10.1371/journal.pgen.0020180

**Published:** 2006-12-29

**Authors:** Mark M Metzstein, Mark A Krasnow

**Affiliations:** Howard Hughes Medical Institute and Department of Biochemistry, Stanford University School of Medicine, Stanford, California, United States of America; Stowers Institute for Medical Research, United States of America

## Abstract

Nonsense-mediated mRNA decay (NMD) is a cellular surveillance mechanism that degrades transcripts containing premature translation termination codons, and it also influences expression of certain wild-type transcripts. Although the biochemical mechanisms of NMD have been studied intensively, its developmental functions and importance are less clear. Here, we describe the isolation and characterization of *Drosophila* “photoshop” mutations, which increase expression of green fluorescent protein and other transgenes. Mapping and molecular analyses show that photoshop mutations are loss-of-function mutations in the *Drosophila* homologs of NMD genes *Upf1, Upf2,* and *Smg1*. We find that *Upf1* and *Upf2* are broadly active during development, and they are required for NMD as well as for proper expression of dozens of wild-type genes during development and for larval viability. Genetic mosaic analysis shows that *Upf1* and *Upf2* are required for growth and/or survival of imaginal cell clones, but this defect can be overcome if surrounding wild-type cells are eliminated. By contrast, we find that the PI3K-related kinase *Smg1* potentiates but is not required for NMD or for viability, implying that the *Upf1* phosphorylation cycle that is required for mammalian and Caenorhabditis elegans NMD has a more limited role during *Drosophila* development. Finally, we show that the SV40 3′ UTR, present in many *Drosophila* transgenes, targets the transgenes for regulation by the NMD pathway. The results establish that the *Drosophila* NMD pathway is broadly active and essential for development, and one critical function of the pathway is to endow proliferating imaginal cells with a competitive growth advantage that prevents them from being overtaken by other proliferating cells.

## Synopsis

Cells possess a variety of surveillance mechanisms that detect and dispose of defective gene products. One such system is the nonsense-mediated mRNA decay (NMD) pathway, which degrades aberrant mRNAs containing nonsense mutations or other premature translation stop signals. In a genetic screen in *Drosophila,* the authors identified a set of mutations they call “photoshop” mutations because they increase expression of green fluorescent protein transgenes such that cells expressing green fluorescent protein are more easily visualized. They found that the photoshop mutations are mutations in three different genes implicated in NMD. Using these mutations, they show that the NMD pathway not only degrades mutant mRNAs but also influences expression of many transgenes and dozens of endogenous genes during development and is essential for development beyond the larval stage. One important function of the pathway is to provide proliferating cells with a competitive growth advantage that prevents them from being overtaken by other proliferating cells during development. Thus, the *Drosophila* NMD pathway has critical cellular and developmental roles beyond the classical surveillance function of eliminating mutant transcripts.

## Introduction

Nonsense-mediated mRNA decay (NMD) is a cellular surveillance pathway in eukaryotes that recognizes and degrades transcripts with premature termination codons (PTCs). Such transcripts arise as a consequence of genomic mutation, as in numerous human genetic diseases [1,2], and from errors in transcription and aberrant RNA splicing. Destruction of PTC-containing transcripts by NMD prevents production of truncated, potentially harmful proteins that can interfere with normal cellular processes (e.g., [3]). The NMD pathway has also been found to influence expression of a variety of wild-type transcripts (reviewed in [4]), implying that the pathway has regulatory roles beyond its surveillance function. In this paper, we describe *Drosophila* mutants that affect NMD.

NMD pathway genes were discovered by genetic studies in yeast (*up-frameshift suppressor [Upf]* genes; [5]) and Caenorhabditis elegans (*suppressor with morphogenetic effect on genitalia [smg]* genes; [6]), and their functions and mechanisms of action have been characterized by molecular genetic and biochemical analysis of the proteins and target RNAs in yeast [7] and cultured mammalian and *Drosophila* cells [8–10]. There are three conserved core components of the pathway, *Upf1 (smg-2), Upf2 (smg-3),* and *Upf3 (smg-4)* (reviewed in [11]). *Upf1* is an RNA helicase that associates with the translation termination complex at PTCs and, at least in yeast, targets the RNA to cytoplasmic RNA processing centers called P bodies [12]. *Upf1* is proposed to recruit *Upf2* and *Upf3* to these termination complexes, which leads to activation of decapping enzymes and nucleases that degrade the target RNA.

Additionally, in metazoans, *Upf1* undergoes a phosphorylation cycle (reviewed in [13]). *Upf1* is phosphorylated on serine residues by Smg1, a PI3K-related kinase. The phosphates are subsequently removed by complex(es) containing Smg5, Smg6, and/or Smg7, three similar proteins that are thought to recruit the phosphatase PPA2. The *Upf1* phosphorylation cycle is apparently necessary for *Upf1* and NMD activity at least in some organisms, because NMD function is abrogated when Smg1, Smg5, Smg6, or Smg7 activity is reduced [6,9,10,14].

One intriguing mechanistic question is how the NMD machinery distinguishes a PTC from a normal termination codon. In mammals, an important feature appears to be the relationship between the termination codon and splice junctions in the mRNA [15]. Most normal termination codons are located beyond the last splice junction, in the final exon of the mRNA. Termination codons that lie upstream of an exon–exon boundary are generally recognized as premature and target the mRNA for destruction by NMD. Such boundaries are marked after splicing by deposition of a multiprotein complex, the exon junction complex (EJC), which includes *Upf2* and *Upf3.* One current model proposes that EJCs along an mRNA are normally all displaced by the translocating ribosome, but if the ribosome encounters a termination codon before the last EJC, this EJC remains and promotes delivery of *Upf2* and *Upf3* to *Upf1* at the termination complex to activate NMD [8,12]. However, this cannot be the sole mechanism of PTC recognition because there are mammalian transcripts with stop codons in the last exon that are subject to NMD, as well as transcripts with stop codons upstream of introns that are resistant to NMD (reviewed in [15]). Furthermore, PTC recognition in yeast and cultured *Drosophila* cells can occur in the absence of introns and splice junctions [7,10]. In yeast it appears that the distance between the stop codon and a special site in or near the 3′ UTR, or the ability of proteins bound at these sites to efficiently associate, marks a termination codon as premature and targets the mRNA for destruction by NMD [16], and this also appears important for NMD targeting of certain mammalian transcripts [17].

Although the mechanism of the NMD pathway has been studied extensively, its developmental functions have received less attention. Yeast and C. elegans NMD pathway mutants are viable as homozygotes and have only subtle or no effects on development and differentiation. The most conspicuous defects in C. elegans mutants are the swollen bursa in the tail of adult males and swollen vulva of hermaphrodites, both of which apparently result from an effect on morphogenesis rather than cell lineage [6]. By contrast, mouse UPF1^−/−^ mutants are not viable [18], and RNA interference knockdown of *Upf1, Smg1,* or other NMD pathway genes in cultured *Drosophila* S2 or mammalian cells causes a cell cycle arrest, implicating the NMD pathway in cell cycle progression [19,20]. However, two recently described mutations in the *Drosophila* homolog of *Smg1,* the only extant mutations in *Drosophila* NMD genes, are homozygous viable and do not appear to affect NMD, raising questions about the function of *Smg1* and the NMD pathway in *Drosophila* [21].

Here, we describe the isolation and characterization of *Drosophila* mutants that enhance expression of green fluorescent protein (GFP) and other transgenes. We demonstrate that these are loss-of-function mutations in three NMD pathway genes, *Upf1, Upf2,* and *Smg1*. We show that *Upf1* and *Upf2* are required for NMD pathway activity, whereas *Smg1* has a variable, gene-selective role potentiating the pathway. We then use the *Upf1* and *Upf2* mutations to characterize the functions and endogenous RNA targets of the pathway in *Drosophila* development. We find that the NMD pathway is broadly active during development and required for proper expression of dozens of endogenous genes and for larval viability, and that one critical function of the pathway is to enhance the ability of cells to compete with other cells during proliferative growth.

## Results

### Identification of Mutations That Increase Transgene Expression in *Drosophila*

**Figure 1:**
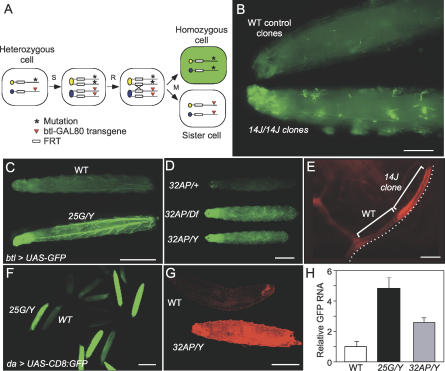
Identification of Photoshop Mutants (A) FLP/MARCM system used to generate GFP-labeled homozygous mutant tracheal cell clones in heterozygous animals. Heterozygous cells have mutations (asterisk) in trans to a *GAL80* transgene (triangle) that inhibits expression of a tracheal-specific, *btl-GAL4*-driven *GFP* transgene on another chromosome (not shown). All cells also carry a heat-inducible FLP transgene on another chromosome (not shown). Following S phase of the cell cycle (S), FLP-mediated recombination (R) at the FRT, and M phase (M), homozygous mutant tracheal cells are segregated that lack the *GAL80* transgene and express GFP (green), distinguishing them from homozygous *GAL80* sister cells and nonrecombined heterozygous cells. (B) Anteriors of L3 larvae with wild-type (WT; *y w*) control clones (top) or photoshop mutant *14J* clones (bottom). Larvae were photographed next to each other to facilitate comparison. Note strong GFP signal in *14J* clones but barely detectable signal in control clones. Bar, 0.5 mm. (C) GFP signal is increased throughout tracheal system in photoshop mutant *25G* larvae. A wild-type *(y w FRT^19A^/Y; btl-GAL4, UAS-GFP)* control larva is shown above, and a hemizygous *25G/Y* photoshop mutant *(25G/Y; btl-GAL4, UAS-GFP)* larva is shown below. Bar, 1 mm. (D) GFP signal is increased throughout tracheal system in photoshop *32AP* larvae. Above is a control heterozygous (*32AP/+*; *btl-GAL4, UAS-GFP*/+) larva showing weak GFP signal throughout tracheal system. In the middle is a *32AP/Df* mutant female (*32AP/Df; btl-GAL4, UAS-GFP*/+) larva showing enhanced GFP signal throughout. Below is a *32AP/Y* mutant male (*32AP*/Y*; btl-GAL4, UAS-GFP*/+) larva: GFP signal is indistinguishable from that observed in a *32AP/Df* larva, indicating *32AP* is an amorphic (null) allele. Bar, 1 mm. (E) Effect of a photoshop mutation on a *DsRed* transgene. Photoshop *14J* tracheal clone in *y w 14J FRT^19A^/ w FRT^19A^, FLP122* larva carrying *btl-GAL4* and *UAS-DsRed1* transgenes. Two unicellular tracheal tubes are indicated by brackets; *14J* ^−^ clone has higher DsRed1 signal than neighboring wild-type (*14J^+^*) cell. Bar, 25 μm. (F) Effect of photoshop *25G* mutation outside the tracheal system. A mixture of wild-type and *25G/Y* mutant animals carrying *da-GAL4, UAS-CD8:GFP* is shown. GFP signal is increased throughout all tissues of the *25G* mutants. *25G* genotype of high-signal larvae was confirmed by PCR. Bar, 2 mm. (G) Effect of photoshop *32AP* mutation in epidermis. A wild-type larva is shown above, and below is a *32AP/Y* mutant larva (bottom) carrying *e22c-GAL4, UAS-nls:DsRed2,* which expresses a nuclear localized DsRed2 in epidermis and other epithelial tissues. Animals were photographed next to each other. The nls:DsRed2 signal is enhanced in the epidermis of the *32AP/Y* mutant. Bar, 1 mm. (H) Effect of photoshop mutants on *GFP* RNA levels. Quantitative real time RT-PCR was done on the *GFP* transcript in wild-type *(y w FRT^19A^/Y), 25G/Y,* and *32AP/Y* L3 larvae carrying one copy of *da-GAL4* and *UAS-CD8:GFP*. Results of replicate reactions were individually normalized to parallel reactions on *rp18LA* transcript. Values shown are mean ± 2 standard errors of the mean normalized to value in wild type.

We conducted a genetic mosaic screen of ethane methyl sulfonate–induced mutations on the X chromosome for tracheal (respiratory) system mutants. Details of the screen will be described elsewhere. In the screen, we used the S. cerevisiase FLP1 recombinase (FLP)/FLP1 recombinase target (FRT) system [22] to generate homozygous mutant cell clones in otherwise heterozygous animals. Tracheal clones were identified by labeling them with GFP using the MARCM system [23] in which a *btl-GAL4* transgene drives tracheal expression of a *UAS-GFP* transgene but is kept off in heterozygous cells by a *btl-GAL80* transgene present on the wild-type X chromosome (Figure 1A). In a screen of 749 ethane methyl sulfonate–induced X-linked lethal lines, we identified six mutations *(13D, 14J, 25G, 26A, 29AA,* and *32AP)* that caused markedly increased GFP signal in homozygous mutant tracheal clones compared to control wild-type clones examined as third instar (L3) larvae (Figure 1B). We named this the “photoshop” phenotype because it increased visualization of clones like that achieved by digital enhancement with Photoshop software (Adobe, http://www.adobe.com). The photoshop phenotype was not dependent on the *btl-GAL80* repressor in the MARCM system: homozygous photoshop tracheal cell clones in *btl-GAL4, UAS-GFP* larvae showed a similar enhancement of GFP signal, and viable hemizygous *25G* and *32AP* larvae and adults (see below) carrying the same transgenes showed increased GFP signal throughout the tracheal system (Figure 1C and 1D and data not shown). The photoshop phenotype was not specific to GFP, because mutant clones showed similar enhancement of DsRed1 signal (Figure 1E). All tracheal cells examined showed the photoshop phenotype, and all six mutations gave similar GFP enhancement.

All but two of the mutations *(14J, 29AA, 13D,* and *26A)* lead to hemizygous male lethality before L3. One exception was *25G: 25G/Y* hemizygous males and *25G* homozygous females developed to L3 larvae at approximately normal frequencies and produced a few percent of escaper adults. These adults appeared morphologically normal but had greatly reduced fertility. The other exception was *32AP,* which after removal of extraneous linked lethal loci (see Materials and Methods) was found to be hemizygous-male and homozygous-female viable and fertile. We generated a deficiency uncovering *32AP* and found that *32AP/Df* showed the same GFP enhancement as *32AP/Y* (Figure 1D), implying that *32AP* is an amorphic (null) allele.

To study the effect of the mutations on GFP signal in other tissues and other stages of development, we used the viable alleles *25G* and *32AP* along with *da-GAL4* and *UAS-CD8:GFP* transgenes, which give ubiquitous expression of GFP [24]. Compared to control animals, *25G*/*Y* male and homozygous *25G* female larvae and adults had greatly increased GFP in all tissues examined, including epidermis, salivary glands, fat body, and eyes (Figure 1F and data not shown). *32AP* mutants also showed enhanced GFP signal in all tissues examined, although enhancement appeared slightly weaker than in *25G* mutants. *32AP* also enhanced levels of a nuclear localized DsRed2 construct expressed in the larval epidermis (Figure 1G). Thus, photoshop mutations increase transgene signal in many, if not all, larval and adult tissues.

To test whether the increased signal seen in the photoshop mutants was due to increased expression of the transgene, we performed quantitative RT-PCR experiments on RNA derived from hemizygous *25G,* hemizygous *32AP,* or control wild-type male L3 larvae, each also containing *da-GAL4* and *UAS-CD8:GFP*. GFP RNA levels were increased ~5-fold in *25G* mutants and ~2.5-fold in *32AP* mutants compared to the wild type (Figure 1H). Hence, the increased GFP signal in photoshop mutants is due at least in part to increased GFP RNA levels.

### Photoshop Mutations Are Loss-of-Function Alleles of Three NMD Pathway Genes

**Figure 2:**
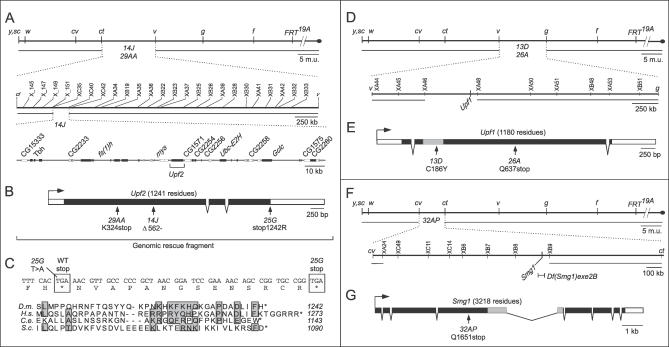
Photoshop Mutations Are Alleles of *Upf2, Upf1,* and *Smg1* (A) Mapping of photoshop mutations *14J* and *29AA*. Shown are genetic maps of the X chromosome (top) and *ct–v* interval (middle) with visible markers (top) and SNP markers (middle) used to map lethality associated with *14J* and *29AA* mutations. Lines beneath maps show regions of *14J* and *29AA* X chromosomes (top) or *14J* chromosome (middle) that were hemizygous-male viable, localizing *14J* between XC35 and XC40 markers. Bottom panel shows predicted genes in mapped interval. Genes are alternately shaded black and white for clarity. (B) *Upf2* mutations in photoshop mutants *14J, 29AA,* and *25G*. *29AA* (AAG to TAG) and *25G* (TGA to AGA) are point mutations that disrupt *Upf2* protein sequence as indicated, whereas *14J* is a deletion of nucleotides 1682–1695 (TCCTGCCCTATCTC) that disrupts the coding sequence at codon 562. Filled boxes, coding sequence; open boxes, UTRs; arrow, direction of transcription; bracket, extent of *Upf2* genomic fragment in rescue transgenes, extending from ~600 bp upstream of *Upf2* mRNA to 78 bp downstream of polyadenylation site. (C) 3′ end of *Upf2* coding sequence showing effect of *25G* mutation, which converts stop to arginine codon and extends coding sequence 15 residues. Below is an alignment of C-termini of *Drosophila melanogaster (D.m.) Upf2* and homologs from human *(H.s.), C. elegans (C.e.),* and *Saccharomyces cerevisiae (S.c.)*. Similar residues shaded grey. (D) Mapping of photoshop mutations **13D** and *26A*. Lethality associated with *13D* and *26A* mutations mapped between markers XA46 and XA48, an interval that includes *Upf1*. (E) Mutations in *Upf1* in *13D* and *26A*. Grey box, region homologous to domain in S. cerevisiae Upf1p required for interaction with Upf2p. (F) Mapping of photoshop mutation *32AP*. Lines beneath each map indicate regions of *32AP* chromosome that did not enhance GFP expression in hemizygous males, showing that *32AP* maps between XA24 and XB9, an interval containing *Smg1*. *Df(Smg1)exe2B* is an ~46-kb deficiency that uncovers *Smg1* and the photoshop phenotype of *32AP*. (G) Mutation in *Smg1* in *32AP*. *32AP* also contains a silent mutation (not shown) in codon 1755 (GCC to GCT). Grey box, Smg1 kinase domain.

We used meiotic recombination to map the lethality and the mosaic tracheal GFP enhancement phenotype of the photoshop mutations. *14J* and *29AA* mapped between *ct* and *v* (Figure 2A). Complementation analysis (see below) showed that *14J* and *29AA* and the semi-lethal allele *25G* formed a single complementation group. *13D* and *26A* mapped between *v* and *g* (Figure 2D), and complemented *14J* for lethality. *32AP* was mapped by its enhancement of GFP expression in hemizygous males, and localized between *cv* and *ct* (Figure 2F). Thus, the six photoshop mutations define at least three loci.

We further refined the position of mutation *14J* by single nucleotide polymorphism (SNP) mapping and localized it to an ~200-kb interval (Figure 2A). DNA sequencing of predicted genes in this interval identified changes in the *Drosophila* homolog of the NMD pathway gene *Upf2* in all three alleles of the *14J* complementation group. The *14J* mutation is a 14-bp deletion approximately halfway through the 1,241-residue coding sequence of *Upf2* (Figure 2B), which causes a frame shift at codon 562 followed by a stop 40 codons later. *29AA* is a nonsense mutation at codon 324. Both *14J* and *29AA* truncate the *Upf2* coding sequence and are likely to be null alleles. A transgene comprising the *Upf2* gene (Figure 2B) rescued *14J* hemizygous males and homozygous females to viability and fertility; *29AA* could not be tested because of a linked lethal mutation. We conclude that *14J* and *29AA* are loss-of-function mutations in *Upf2*.

*25G* hemizygous males and homozygous females survived to L3 and beyond (see above), whereas *25G/14J* trans heterozygotes were not viable after L2, suggesting that *25G* is a hypomorphic allele of *Upf2*. The *Upf2^+^* transgene rescued *25G* hemizygous males and homozygous females to viability and fertility, confirming this assignment. The *25G* allele is a curious mutation that alters the natural *Upf2* stop codon to an arginine codon (TGA to AGA). The next in-frame termination codon is 45 bp downstream, so *25G* encodes a Upf2 protein with a 15-residue C-terminal extension (Figure 2C). The C-terminus of yeast Upf2p is required for its interaction with Upf1p [25], so the Upf2 extension might interfere with this function. The *25G* mutation also creates a consensus 3′ splice site, but RT-PCR experiments on *25G* mutant RNA did not detect any novel splice forms involving this site.

The map positions of *13D* and *26A* were refined using SNP markers, and the lethality associated with each allele localized to an ~400-kb interval that contained the *Drosophila* homolog of *Upf1* (Figure 2D). Sequencing of the *Upf1* gene in *26A* revealed a nonsense mutation (CAG to TAG; Q637stop) in the middle of the 1,180-residue coding sequence (Figure 2E), and sequencing of *13D* identified a missense mutation (TGC to TAC) that alters a conserved cysteine (C186Y) in a domain required for interaction between yeast Upf1p and Upf2p [25] (Figure 2E). We also found that *26A* and *13D* failed to complement each other for lethality. Thus, *26A* and *13D* appear to be loss-of-function alleles of *Upf1,* and we refer to them as *Upf1^26A^* and *Upf1^13D^*.

*32AP* was mapped to a 2-Mb interval containing 122 predicted genes, one of which is the *Drosophila* homolog of *Smg1* (Figure 2F). We generated an ~46-kb deficiency that removes ten genes including *Smg1,* and found that this deficiency failed to complement the photoshop phenotype of *32AP* (Figure 1D). DNA sequencing identified a nonsense mutation (CAG to TAG) in codon 1651 of the 3,218-residue *Smg1* coding sequence, which truncates the Smg1 protein before most of the conserved domains, including the kinase domain (Figure 2G). Thus, *32AP* is most likely a null *Smg1* allele, consistent with the gene dosage experiments described above (Figure 1D). We refer to it as *Smg1^32AP^*.

### Photoshop Mutations Abolish or Reduce NMD of a Mutant Transcript In Vivo

**Figure 3:**
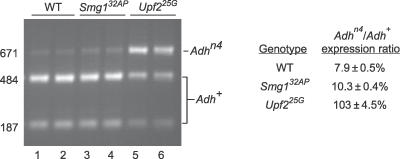
Effect of Photoshop Mutations on Steady State Levels of an *Adh* Transcript Containing a Nonsense Mutation Left, agarose gel analysis of PvuII-digested products of RT-PCR of *Adh* RNA extracted from heterozygous *Adh^n4^/Adh^+^* adult males that were wild-type (WT) for NMD genes (lanes 1 and 2), hemizygous for *Smg1^32AP^* (lanes 3 and 4), or hemizygous for *Upf2^25G^* (lanes 5 and 6). *Adh^n4^* is a nonsense mutation that also disrupts a PvuII site that divides the 671-bp PCR product into 484-bp and 187-bp fragments. For each genotype, two independent RNA samples were analyzed. Right, quantification of *Adh^n4^* and *Adh^+^* RNA levels by capillary electrophoresis of PvuII-digested RT-PCR products. Similar results were obtained by directly sequencing the same RT-PCR products and quantitating the level of each product from the chromatogram.

*Upf1, Upf2,* and *Smg1* are required for NMD in yeast and C. elegans and have been shown to be involved in NMD in cultured *Drosophila* cells [10], although recent data question the in vivo role of *Smg1* in *Drosophila* [21]. To determine whether photoshop genes are required for NMD in vivo, we tested the effects of photoshop mutations on mRNA levels of *Adh^n4^,* a nonsense mutation in *Adh* [26] that subjects the mRNA to NMD in S2 cells [10]. We isolated RNA from adult wild-type males (*y w FRT^19A^/Y; Adh^n4^/Adh^+^*) and from *Upf2* (*Upf2^25G^/Y; Adh^n4^/Adh^+^*) and *Smg1* (*Smg1^32AP^/Y; Adh^n4^/Adh^+^*) mutant males, amplified *Adh* mRNA by RT-PCR, and quantitated the *Adh^+^* and *Adh^n4^* products to assess the relative levels of the two transcripts (Figure 3). In wild-type animals, steady state transcript levels from the *Adh^n4^* allele were reduced 13-fold relative to *Adh* ^+^ transcripts, presumably because of increased turnover of the mutant transcript by the NMD pathway. In *Upf2^25G^* animals, *Adh^n4^* levels increased 13-fold, such that *Adh^n4^* and *Adh^+^* transcript levels were equivalent, implying that the *25G* mutation abolishes NMD pathway function. By contrast, *Smg1^32AP^* had only a modest effect, increasing *Adh^n4^* levels by 30%.

### The NMD Pathway Targets the SV40 3′ UTR of *Drosophila* Transgenes

**Figure 4:**
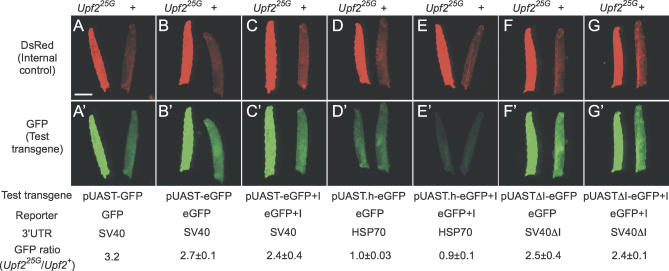
Effect of a Photoshop Mutation on Expression of GFP Transgenes Pairs of larvae of genotypes *Upf2^25G^/Y; e22c-GAL4, UAS-nls:DsRed2/UAS-GFP* (*Upf2^25G^* mutant, left in each panel) and *w/Y; e22c-GAL4, UAS-nls:DsRed2/UAS-GFP* (*Upf2^+^* control, right in each panel). The *DsRed2* (internal control) transgene contains an SV40 3′ UTR and was the same in all larvae, whereas the *GFP* test transgene differed in reporter and 3′ UTR sequences as indicated. DsRed channel (A–G) shows effect of *Upf2^25G^* on the internal control *DsRed2* transgene. GFP channel (A′–G′) shows effect of *Upf2^25G^* on the *GFP* test transgenes indicated. Photographic exposures were the same and images were processed identically to facilitate comparison. Similar results were obtained for at least two independent insertions of each *eGFP*-variant transgene. The ratio of GFP expression for each transgene in *Upf2^25G^* versus *Upf2^+^* larvae was quantitated by GFP fluorescence measurements of micrographs of pairs of *Upf2^25G^* and *Upf2^+^* larvae, and the fluorescence ratio (average ± standard deviation, *n* = 2 independent insertions for each *eGFP* transgene) is shown. (The expression ratio of the *DsRed2* internal control construct in *Upf2^25G^* versus *Upf2^+^* larvae was 4.7 ± 0.7). Only *GFP* transgenes with an SV40 3′ UTR were enhanced in *Upf2^25G^* mutants, independent of the intron in this UTR. Transgenes with a 3′ UTR derived from *hsp70* were not enhanced. eGFP, enhanced GFP reporter; + I, with added synthetic intron; ΔI, SV40 intron deleted; pUAST.h, pUAST with *hsp70* 3′ UTR replacing SV40 3′ UTR. Bar, 1 mm.

To investigate how the NMD genes influence transgene expression, we examined the effect of photoshop mutations on transgenes containing different reporter genes, promoters, and 3′ UTRs (Table 1; Figure 4). All of the transgenes whose steady state expression levels were found to be upregulated in photoshop mutants were constructed in the *pUAST* vector, the standard vector for gene misexpression in *Drosophila* [27]. This vector contains multiple binding sites for the yeast transcription factor GAL4 followed by a core promoter derived from the *hsp70* gene upstream (5′) of the reporter insertion site, and the SV40 3′ small t antigen intron and polyadenylation signal (henceforth referred to as the SV40 3′ UTR) downstream (3′) of the insertion site [28]. Hence, it was possible that the photoshop phenotype was due to increased GAL4 expression or activity, increased *hsp70* promoter activity, an interaction with the SV40 3′ UTR, or some special feature of the GFP and DsRed coding sequences. *Upf2^25G^* enhanced expression of the *pUAST-eGFP* transgene (Figure 4B), which has a metazoan optimized codon bias, suggesting that the unusual codon bias of native GFP and DsRed genes is not critical for the photoshop effect. The GFP and DsRed reporters do not contain introns in their coding sequences, so we inserted a 61-bp intron from *Drosophila* gene CG3585 into *pUAST-eGFP* to make *pUAS-eGFP+I*. This transgene did not show significantly increased eGFP signal in wild type and was still sensitive to photoshop mutations (Figure 4C), suggesting that the photoshop effect is not through surveillance of intronless coding regions.

**Table 1:**
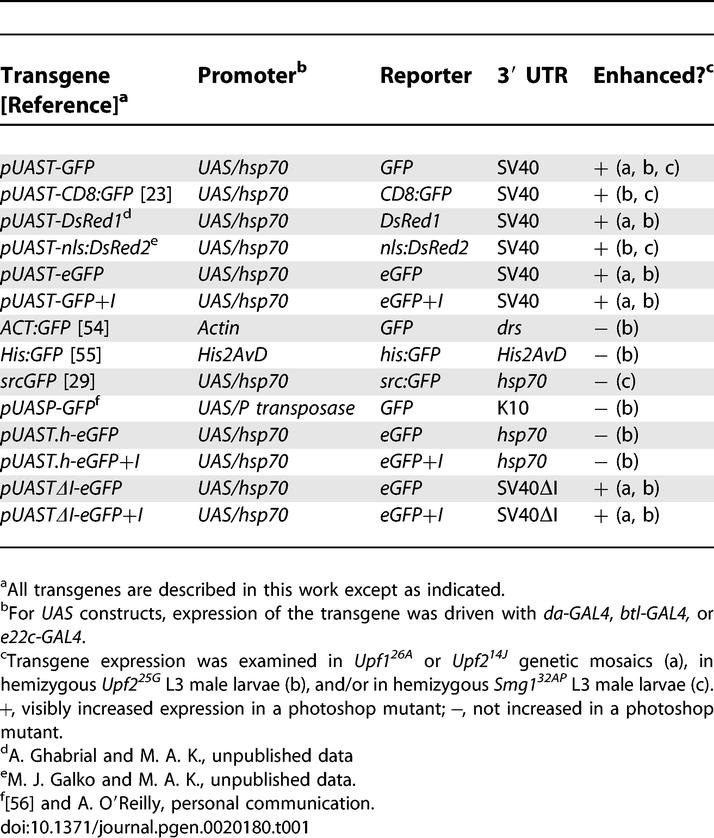
Transgenes Tested for Enhanced Expression in Photoshop Mutants

One tested transgene, *src:GFP,* which was not upregulated in *Smg1^32AP^* animals, contains the same GAL4 binding sites and *hsp70* promoter as the photoshop-sensitive *pUAST* constructs, but its 3′ UTR and polyA signal are derived from *Drosophila hsp70* instead of SV40 [29]. This implicated the SV40 3′ UTR in the photoshop effect. To test the role of the SV40 3′ UTR directly, we replaced the SV40 3′ UTR in *pUAST-GFP* and *pUAST-GFP+I* constructs with the *hsp70* 3′ UTR. Expression of these constructs was insensitive to photoshop mutations (Figure 4D and 4E), confirming the importance of the 3′ UTR. Interestingly, expression levels of GFP from the constructs containing the *hsp70* 3′ UTR were somewhat lower than those of the corresponding *pUAST-eGFP* or *pUAST-eGFP+I* transgenes in a wild-type background and much lower than those of *pUAST-eGFP* or *pUAST-eGFP+I* transgenes in a photoshop mutant background. The significance of this finding is discussed below.

A small intron present in the SV40 3′ UTR was an appealing candidate for sensitizing transcripts to the photoshop effect. A model for recognition of PTCs in vertebrates is that they occur 5′ of the site of an intron, the same arrangement of the termination codons of GFP and DsRed with respect to the SV40 intron. RT-PCR experiments confirmed that the SV40 intron was indeed recognized and spliced in *Drosophila* larvae. However, deletion of this intron in the *pUAST-eGFP* and *pUAST-eGFP+I* constructs (to make *pUASTΔI-eGFP* and *pUASTΔI-eGFP+I*) did not eliminate the photoshop effect (Figure 4F and 4G). Thus, targeting of the SV40 3′ UTR by the NMD pathway does not require splicing of this intron, the only known intron in the UTR.

### Targets of the NMD Pathway during Development

To identify candidate endogenous targets of the NMD pathway*,* we compared steady state RNA levels in hemizygous *Upf2^25G^* male larvae to those in wild-type male larvae using a whole genome microarray. To avoid confounding effects of the *Upf2* mutation on developmental progression, we focused our analysis on a set of 954 genes that do not undergo significant changes in expression levels during development or differ in expression between male and female larvae (see Materials and Methods). Among this set, we found 14 genes whose expression was upregulated 2-fold or more, and 26 genes that were downregulated 2-fold or more in the mutant compared to wild type (Tables S1 and S2). Genes whose expression was downregulated could be novel targets whose expression is paradoxically enhanced by the NMD pathway, or they could be indirect effects of NMD pathway inactivation. The affected genes encode proteins of diverse classes, including signal transduction molecules, proteases, and proteins involved in cell metabolism. Most of the affected genes differed from ones identified recently in *Drosophila* S2 cells depleted of NMD-gene function by RNA interference [19], suggesting tissue-specific regulation.

**Figure 5:**
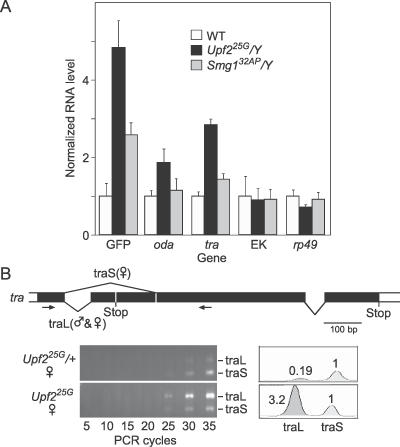
Effect of Photoshop Mutations on Expression of Endogenous Genes (A) Results of quantitative real-time RT-PCR of indicated genes using RNA from *y w FRT^19A^/Y* (WT), *Upf2^25G^/Y,* and *Smg1^32AP^/Y* L3 larvae carrying one copy of *da-GAL4* and *UAS-CD8:GFP*. Amplifications of replicate reactions were individually normalized to internal control reactions with *rp18LA*. Values shown are means ± 2 standard errors of the mean relative to the results with *y w FRT^19A^/Y*. GFP RNA levels in these larvae (see Figure 1H) are included for comparison. EK, photoshop-independent control gene defined by expressed sequence tag EK161155. (B) Effect on *tra* RNA levels in females. Above are shown sex-specific splice patterns in the coding portion of the *tra* transcript. Males use splice pathway shown below line to produce traL, whereas females use both splice pathways to produce traL and traS. traS encodes a 197-residue protein, whereas traL contains an early termination codon and encodes a 37-residue protein. Arrows, position of PCR primers used to simultaneously amplify traL (364-bp product) and traS (189 bp). Bottom left shows RT-PCR on RNA from heterozygous *Upf2^25G^/+* and homozygous *Upf2^25G^* female larvae; aliquots of reaction were taken at PCR cycles indicated. Note increased traL in *Upf2^25G^* homozygotes. Bottom right shows quantification of results after 30 PCR cycles. Areas of the peaks are indicated, normalized to traS peak. These and similar experiments (not shown) indicate traL is increased ~10-fold in *Upf2^25G^* mutants. This was greater than the value measured for *Upf2^25G^* mutant males (~4-fold; Table S1; Figure 5A), perhaps due to sex-specific differences in NMD or differences in sensitivity of the assays.

Two well characterized genes upregulated in the photoshop mutant were analyzed further. The mRNA of *orthinine decarboxylase antizyme (oda,* also called *gut feeling)* contains a naturally occurring coding sequence frame shift [30] that causes the transcript to contain early termination codons, and is an NMD target in cultured *Drosophila* S2 cells [19]. *oda* transcript is also a target of the NMD pathway during development: *oda* RNA levels were increased in hemizygous *Upf2^25G^* larvae as determined by microarray analysis (3-fold) and by quantitative RT-PCR (2-fold; Figure 5A). However, *oda* transcript levels were not significantly increased in *Smg1^32AP^* mutants (Figure 5A).

The sex determination gene *transformer (tra)* [31] is also an NMD pathway target during development, as in S2 cells [19]. *tra* transcript levels were increased 3- to 4-fold in hemizygous *Upf2^25G^* larvae, as determined by microarray analysis and quantitative RT-PCR (Figure 5A). *Smg1^32AP^* also increased *tra* levels, but to a lesser extent (Figure 5A). *tra* might be expected to be an NMD pathway target because the primary transcript is spliced in males to produce a long mRNA (traL) that contains an early termination codon that would likely be recognized as premature, whereas in females some of the primary transcript is alternatively spliced to produce a shorter transcript (traS) lacking the early termination codon and encoding full-length protein (Figure 5B). Indeed, RT-PCR experiments indicate that the increase in *tra* levels in *Upf2^25G^* mutant males and females was due to selective stabilization of traL (Figure 5B and data not shown).

### *Drosophila* NMD Genes Are Dispensable for Many Developmental Processes but Provide Cells a Competitive Growth or Survival Advantage

**Figure 6:**
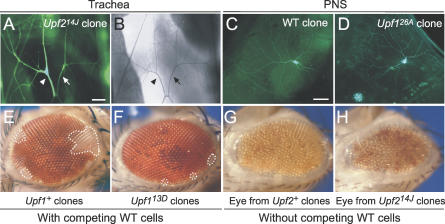
Effects of Photoshop Mutations on Tracheal System, Nervous System, and Eye Development (A and B) Fluorescence (A) and brightfield (B) images of tracheal dorsal branch terminal cells in *y w Upf2^14J^ FRT^19A^/y w FRT^19A^, FLP122; btl-GAL4, UAS-GFP/+* mosaic L3 larva. Homozygous *Upf2^14J^* clone (arrowhead) expresses GFP at a higher level than contralateral heterozygous control cell (arrow), but clone has formed normal cytoplasmic branches (A) and a normal air-filled lumen in each branch (B). Bar in (A) (for [A] and [B]), 25 μm. (C and D) Wild-type (WT) control clone (C) and homozygous *Upf1^26A^* clone (D) in type IV da neurons labeled with *ppk-GAL4, UAS-CD8:GFP*. Both control and *Upf1^26A^* clones show complex dendritic arborization fields typical of type IV da neurons, although in the latter the aborizations are easier to visualize because of increased GFP expression. Bar in (C) (for [C] and [D]), 100 μm. (E and F) Control (*y w*) wild-type clones (E) and  *Upf1^13D^* clones marked with *w^−^* in adult eyes. Wild-type clones (E) are easily detected as *w^−^* patches (white areas, demarked with dotted lines) in the *w^+^* (red) heterozygous eye, whereas only small *w* ^−^ clones or single *w* ^−^ cells are detected in the *Upf1^13D^* heterozygous eye (F). Similar results were obtained with *Upf2^14J^* (not shown). (G and H) Similar experiment with *Upf2^14J^* mutant except eye cells not part of the clone were eliminated by GMR-*hid* technique. *Upf2^+^* control clones proliferate (G) to form an eye (slight eye roughness is common with GMR-*hid* technique), as do *Upf2^14J^* clones (H), although the latter are somewhat smaller and rougher than the controls. Similar results were obtained with *Upf1^26A^* and *Upf1^13D^* (not shown).

The above results show that the core NMD gene *Upf2* is broadly active during development and influences expression of dozens of genes, including a key sex determination gene, and is required for larval viability. To identify specific cellular and developmental functions of the NMD pathway, we analyzed the effect of photoshop mutations on sex determination and cell growth and differentiation. We did not detect any defects in sex determination in adult males hemizygous for *Upf2^25G^,* despite the observed increase in traL levels: sex-specific splicing of downstream gene *dsx* was normal, as was that of the sex determination genes *Sxl* and *msl-2;* sex combs and genitalia appeared normal; and males made sperm and were capable of mating. Homozygous *Upf2^25G^* females also appeared normal. We also did not detect any defects in larval cell differentiation. Larval tracheal cell clones lacking *Upf1* or *Upf2* displayed wild-type morphology at all levels of branching, including tracheal terminal cell clones, which showed normal branching patterns and luminal structures (Figure 6A and 6B). Likewise, type IV da neurons, another morphologically complex cell type [32], appeared normal when homozygous for *Upf1* or *Upf2* null mutations (Figure 6C and 6D and data not shown).

The only prominent defect we detected in cell clones mutant for NMD genes was an inability to contribute to adult (imaginal) structures. Whereas homozygous clones of all photoshop mutants were readily obtained in the larval tracheal system, we did not recover clones in the adult tracheal system, which is generated by proliferation of imaginal tracheal precursor cells during metamorphosis. We also did not recover large clones in adult epidermis or eyes, although small peripheral eye clones were occasionally observed (Figure 6E and 6F). However, when the GMR-*hid* technique [33] was used to eliminate all heterozygous and wild-type cells in the developing eye, we found that the remaining photoshop mutant cells could proliferate, differentiate, and form an eye, although the eyes were smaller and more disorganized (rougher) than those of controls (Figure 6G and 6H). These results suggest that *Drosophila* NMD genes are not required for cell proliferation, survival, or differentiation, but provide proliferating cells with a competitive growth or survival advantage during development.

## Discussion

We have isolated to our knowledge the first mutations affecting NMD in *Drosophila* based on their ability to enhance expression of a *GFP* transgene, an effect we call the photoshop phenotype. Mapping of the mutations, complementation tests, and molecular analysis demonstrate that the photoshop mutations identify three genes, the *Drosophila* orthologs of NMD pathway genes *Upf1, Upf2,* and *Smg1*. The results show that *Upf1* and *Upf2* are essential genes, required for NMD and, at least in the case of *Upf2*, for proper expression of dozens of native mRNAs during development, including *oda* and the sex-nonspecific form of *tra* that contain early termination codons. By contrast, *Smg1* is dispensable and only potentiates the NMD pathway. Genetic mosaic analysis of the *Upf* genes showed that they are not required for cell proliferation, survival, or complex cell differentiation events such as tracheal and neuronal growth and sprouting, but they provide proliferating imaginal cells with a competitive growth or survival advantage during development. We also mapped the *cis*-acting signal that confers sensitivity to the NMD pathway in the transgenic reporter assay, and discovered that it resides in the heterologous 3′ UTR present in the reporter construct. Below, we discuss the implications of these results for our understanding of the functions and mechanism of the *Drosophila* NMD pathway during development, and compare and contrast them with what has been found for NMD pathway function in other organisms.

### Roles of NMD Genes in *Drosophila* Development

The finding that mutations in *Drosophila* NMD genes *Upf1* and *Upf2* cause lethality during larval development contrasts with the minor effect of mutations in the homologous genes in yeast (mutations have almost no discernable effect on growth or survival [5]) and in C. elegans (mutants are viable and have only morphogenetic defects late in development [6]). Why are *Upf1* and *Upf2* essential in *Drosophila*? One possibility is that they are required to eliminate mutant transcripts with PTCs that encode truncated, deleterious protein products. Such PTC-containing alleles could be present in the background of our *Upf1* and *Upf2* mutants, and in the absence of NMD activity, these mutations become lethal. However, all our *Upf1* and *Upf2* mutations were independently isolated, and the lethality in these lines segregates with the *Upf* mutations, not with other genomic regions. Thus, if this explanation is correct, there would have to be multiple, potentially lethal PTC mutations distributed throughout the genome.

A second possibility is that the NMD pathway has a more general surveillance function that also eliminates naturally occurring transcripts resulting from aberrant splicing events or repetitive DNA elements, and accumulation of such transcripts is toxic. However, many aspects of cell biology and development appear normal in *Upf* mutants. Indeed, sensitive assays examining individual tracheal cells and neurons show that loss of *Upf* function does not lead to cell death or impairment of complex cell morphogenesis events, implying that NMD inactivation does not cause general cellular toxicity.

A third possibility, which we favor, is that the NMD pathway modulates the activity of specific native transcripts, whose misregulation leads to lethality. An initial microarray survey identified several dozen genes of diverse functional classes whose expression was altered in NMD mutant larvae (Tables S1 and S2). Some of the affected transcripts contain early stop codons that are interpreted as bona fide PTCs, as for *tra* and *oda* genes (Figure 5A); other affected transcripts could harbor other *cis*-acting signals that are interpreted as aberrant by the NMD machinery, like the SV40 3′ UTR discussed below. In the absence of the NMD pathway, overexpression of such transcripts would perturb the development or function of select cells or tissues and lead to lethality. Indeed, although many cells appear to develop normally in photoshop mutants, we never observed *Upf1* ^−^ and *Upf2* ^−^ clones in adult tracheae or epidermis, and only small clones were found in the eye, implying that the NMD pathway promotes growth or survival of the proliferating imaginal cells that give rise to these tissues. Because *Upf* genes are broadly expressed [34] and broadly active (Figure 1F and 1G and unpublished data) throughout development, identification of the tissue focus of *Upf* lethality will be an important first step towards identifying the critical cellular targets of NMD gene regulation.

The sole cellular defect we identified in NMD mutants was the absence or small size of mutant clones in the adult tissues described above, which is reminiscent of the cell cycle arrest observed in cultured *Drosophila* S2 cells depleted of *Upf* function [19]. Although these results suggest a function for the NMD pathway in cell proliferation or survival, the requirement for the pathway in these processes is not absolute: *Upf1* ^−^ and *Upf2* ^−^ cells were able to proliferate and form an eye when competing wild-type eye progenitor cells were eliminated (Figure 6). This implies that the NMD pathway provides proliferating imaginal cells with a competitive growth advantage that prevents them from being overtaken by other proliferating cells during development. This could be a cell autonomous effect, like *Minute* mutations [35], or a cell nonautonomous effect, e.g., if the pathway influences expression of a signal secreted from proliferating cells that affects growth of neighboring cells (e.g., [36]). The NMD pathway may play a similar, or more extreme, role in mice, because a mouse UPF1 knockout is lethal and attempts at establishing UPF1^−/−^ embryonic stem cells were unsuccessful [18].

Recently, it has been suggested that *Upf1* and *Upf2* participate in other aspects of gene regulation besides NMD, such as stimulating translation [37] and in translational termination [38] (reviewed by [39]). It is important to note that for neither mouse nor our *Drosophila* mutants is it established that the lethality associated with *Upf1* and *Upf2* mutations derives from their roles in NMD. Indeed, our analysis of *Upf2^25G^* suggests the opposite possibility. This allele appears completely compromised for NMD, as assessed by expression of an *Adh* mRNA carrying a PTC (Figure 3), yet unlike *Upf2* null alleles, some *Upf2^25G^* mutants survived to adulthood. Thus, either the essential function of *Upf2* is in a process other than NMD, or this allele retains residual NMD function sufficient for regulation of its essential target genes but not of others like the mutant *Adh* mRNA.

The differing molecular, cellular, and developmental requirements for NMD- pathway genes in yeast, *C. elegans, Drosophila,* and mice make clear that the function of this pathway has diversified during evolution. Perhaps the ancestral function of the pathway was in some general process like translation termination, and only later did the pathway evolve roles in monitoring transcripts for PTCs and more specialized regulatory roles. Alternatively, the ancestral function could have been regulation of RNAs involved in a specific cellular process such as cell growth regulation, and only later did the pathway acquire a more general role in RNA surveillance.

### *Smg1* Potentiates the *Drosophila* NMD Pathway

Our genetic analysis demonstrated a striking difference in the developmental requirements of *Smg1* compared to those of *Upf1* and *Upf2*. First, *Upf1* and *Upf2* are essential genes, whereas an amorphic *Smg1* allele resulted in viable and fertile animals. Second, a *Upf2* mutation abolished NMD of an *Adh* PTC allele, whereas the amorphic *Smg1* mutation only modestly reduced NMD efficiency. Third, the magnitude of the *Smg1* mutant effect differed at different targets. At some targets, such as *oda,* there was little or no effect of the *Smg1* mutation, whereas at other targets, such as *tra* and a *GFP* transgene, the *Smg1* mutant effect was up to half that of the *Upf2* mutant. The small and gene-selective effect of *Smg1* could explain why a recent genetic analysis failed to detect a role for *Drosophila Smg1* in NMD [21], whereas earlier *Drosophila* cell culture studies suggested an important role for the gene [10]. The small and gene-selective function of *Drosophila Smg1* contrasts with genetic results in *C. elegans,* which did not identify differences in the requirements of *smg-1* and the *Upf1* and *Upf2* homologs *smg-2* and *smg-3* [6]. One possibility is that *Drosophila* has another protein with activity similar to that of Smg1. This seems unlikely because *Smg1* is the only sequence ortholog of *Smg1*-family genes in the *Drosophila* genome, although there are other genes that encode proteins with PI3K-related kinase domains. Another possibility is that phosphorylation of Upf1 by Smg1 is not absolutely required for Upf1 activity in *Drosophila* but only enhances its activity or reactivates spent protein after a catalytic cycle. This would be more similar to the NMD pathway in yeast, which lacks a Smg1 ortholog and is thought to function without a Upf1 phosphorylation cycle, than to the NMD pathways in C. elegans and vertebrates, where the Upf1 phosphorylation cycle is thought to be essential for pathway activity**.**

### Targeting of a Specific 3′ UTR by the *Drosophila* NMD Pathway

We found that a variety of reporter constructs in the *pUAST* transformation vector were upregulated when NMD pathway function was abrogated (Table 1), implying that transcripts derived from this vector are recognized as aberrant by the RNA surveillance machinery. Strictly speaking, this is not an NMD process, because all of the transgenic constructs contain full-length coding sequences with no PTCs. However, because multiple NMD genes are involved in this regulation, and because the observed increase in reporter activity is associated with increased reporter mRNA, it suggests that an NMD-related RNA decay process normally limits expression of *pUAST* transgenes in *Drosophila*. The signal that targets transgenes for regulation by the NMD pathway appears to lie in the SV40 3′ UTR of the *pUAST* vector: all reporter constructs that were sensitive to the NMD pathway contain this UTR, and swapping it for one derived from the *hsp70* gene rendered the transcript insensitive to NMD, the first example to our knowledge of a change in NMD pathway sensitivity due solely to swapping intact 3′ UTRs. Transgenes containing three other endogenous 3′ UTRs (*K10, drs,* and *His2AvD;* see Table 1) were also insensitive to NMD, supporting the conclusion that the SV40 3′ UTR harbors a critical targeting element. Although we have not identified the specific sequence or structural characteristic (e.g., length [17,40]) within the SV40 3′ UTR responsible for targeting by NMD machinery, it does not require the small, naturally occurring intron within the UTR, nor does it require any specific sequences in the translated region of the targeted mRNA. We conclude that a 3′ UTR can provide a critical signal for regulation by the NMD pathway in *Drosophila,* as has been observed in yeast [7] and humans [17].

**Figure 7:**
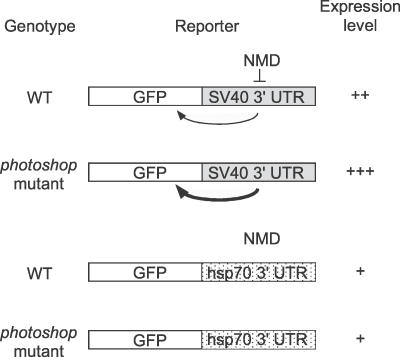
Model for *Drosophila* NMD Pathway Action on SV40 3′ UTR In wild type (WT), SV40 3′ UTR enhances transgene expression (arrow). However, effect is partially offset by transcript degradation by NMD machinery, giving an intermediate level of expression ( +  + ). In photoshop (NMD) mutants, transcript degradation is abrogated, resulting in strong enhancement by SV40 3′ UTR (thick arrow) and high level of expression ( +   +  +  ). The *hsp70* 3′ UTR does not enhance expression (or alternatively promotes degradation) but is also not a target of the NMD pathway, giving a low level of expression (+) insensitive to photoshop mutants.

Our analysis also indicates that the SV40 3′ UTR has a second, positive effect on transgene expression in *Drosophila,* similar to one noted in insect cell culture [41]. In wild-type *Drosophila,* this positive effect is partially offset by destruction of the RNA by the NMD pathway, resulting in an intermediate level of reporter expression (Figure 7). However, in NMD pathway mutants, the positive effect of the SV40 3′ UTR is unmasked, resulting in enhanced reporter expression and the photoshop phenotype. This contrasts with results for transgenes carrying the 3′ UTR from *hsp70,* which does not strongly enhance transgene expression and is not targeted by the NMD pathway, resulting in a lower and photoshop-mutation-insensitive level of reporter expression.

Because expression of UAS-GFP and other reporters is affected by mutations in homologs of all three NMD genes on the chromosome we screened, the assay can likely be used to identify and characterize additional NMD pathway genes on other chromosomes. The assay has two important features. First, because it can be carried out in single cells in genetically mosaic animals, a requirement of candidate NMD genes for organismal viability can be bypassed. Second, the assay is very sensitive to perturbations in the NMD pathway. For example, loss of *Smg1* activity leads to only a modest increase in stability of PTC-containing transcripts but a readily detectable enhancement of GFP reporter expression. Together these features suggest that the transgenic assay system can be used to test requirements in vivo of candidate NMD genes and drugs that influence pathway activity, which could be useful in modulating expression of human disease genes carrying PTCs [42,43].

## Materials and Methods

### Fly stocks and genetics.

*GAL4/UAS* system [27] drivers used were *btl-GAL4* [44], *e22c-GAL4* [45], *ppk-GAL4* [32], and *da-GAL4* [24]. *GFP* and *DsRed* transgenes are referenced in Table 1. *Df*(*Smg1)exe2B* was generated by using FLP-mediated recombination between the FRTs in *P{XP}C3G[d00589]* and *PBac{WH}CG3044[f02328]* as described in Thibault et al. [46]. Marker mutations and balancer chromosomes are described at http://www.flybase.org. Flies were reared at 25 °C on cornmeal/dextrose medium.

The photoshop mutations were obtained by mutagenesis of an isogenized *y w FRT^19A^* chromosome [22] with 25 mM ethane methyl sulfonate overnight [47] in a tracheal mutant screen (to be described elsewhere). The mutations used were on this chromosome unless otherwise noted. To generate homozygous mutant clones, 2- to 6-h-old embryos were collected at 25 °C from a cross of y w * FRT^19A^/FM7 females to *gal80 FRT^19A^, hsFLP122/Y; btl-GAL4, UAS-GFP* males. After a 45-min heat shock at 38 °C to induce FLP expression, embryos were returned to 25 °C to continue development. L3 larvae of genotype y w * FRT^19A^/gal80 FRT^19A^, hsFLP122; btl-GAL4, UAS-GFP/+ were identified by GFP mosaicism within the tracheal system and scored for the photoshop phenotype.

The original *Smg1^32AP^* chromosome (designated *32AP*†) carried lethal mutations not associated with the photoshop phenotype. Lethals were removed by crossing *32AP*†/*y w FRT^19A^* females to *w/Y; btl-GAL4, UAS-GFP* males and identifying L3 larvae with enhanced GFP throughout their tracheal systems. These *Smg1^32AP^/Y; btl-GAL4, UAS-GFP/+* larvae developed into viable adult males. We also found a viable wing morphology mutation on *32AP*† that is allelic to *wavy*. Existing *wavy* alleles do not show a photoshop phenotype, and the wing phenotype is separable from the photoshop phenotype, so *wavy* does not seem to contribute to the photoshop phenotype.

The *Upf2^29AA^* chromosome carries a linked lethal mutation. When recombined away from *Upf2^29AA^,* the mutation had no effect on tracheal development or reporter expression. However, we have not obtained a recombinant containing *Upf2^29AA^* without the extraneous mutation.

For complementation tests of *Upf2,* we used genomic rescue transgenes located on the autosomes to generate males of genotype *y w Upf2^14J^ v g f FRT^19A^*/*Y; P{w^+^, Upf2^+^}/+* and crossed these to y w * FRT^19A^/FM7c females, where the asterisk indicates the tested mutation. Absence of Bar^+^, white-eyed female progeny indicated failure to complement. For complementation tests of *Upf1,* we used the Y-linked duplication *Dp(1;Y)BSC1, y^+^*, which covers the *Upf1* locus. Males of genotype *Upf1^13D^*/*Dp(1;Y)BSC1, y^+^* were crossed to *Upf1^26A^*/*FM7c* females, and the absence of female Bar^+^ progeny indicated a failure to complement.

### Mapping of photoshop mutations.

Identification of SNPs, construction of the SNP map of the X chromosome, and details of their use in mapping X chromosome mutations will be described elsewhere. Briefly, the location of the lethality associated with a photoshop mutation was mapped by crossing y w * FRT^19A^/*sc cv ct v g f FRT^19A^* females (where the asterisk indicates the lethal mutation) to *FM7c/Y* males and scoring the viable male progeny for the visible markers to determine the lethal interval. To refine the map position, we collected male progeny in which a recombination event had occurred within the mapped interval and scored them for SNPs. For each mutation we typically scored 300–400 males for SNPs. For *Upf1^26A^* and *Upf1^13D^,* which map between *v* and *g,* we crossed the y w * FRT^19A^/*sc cv ct v g f FRT^19A^* females to males of genotype *Df(1)64c18, g^1^ sd^1^/Dp(1;2;Y)w^+^* to distinguish *y sc^+^ w cv^+^ ct^+^ v^+^ g f* recombinants, which we could not otherwise identify because of epistasis of *w* over *v* and *g*. We also crossed the y w * FRT^19A^/*sc cv ct v g f FRT^19A^* females to *sc cv ct v g f FRT^19A^/Y* males to identify recombinant females, which were then tested for the photoshop phenotype in genetic mosaics to confirm that the lethality and photoshop phenotype mapped to the same interval. For the viable mutation *32AP,* we followed a similar strategy as for the lethals, except we scored recombinant males for the presence or absence of *32AP* by testing the enhancement of GFP in *btl-GAL4, UAS-GFP* transgenic animals.

### Transgene construction.

For the *Upf2* genomic rescue construct, *Drosophila* BAC 24A2 [48], which contains *Upf2,* was transformed into Escherichia coli strain EL250, which harbors heat-shock-inducible homologous recombination machinery [49]. We then cloned a 200-bp fragment located upstream, and a 300-bp fragment located downstream, of *Upf2* coding sequence and UTRs based on the cDNA RE04053 (rather than the canonical *Upf2* cDNA SD07232, which appears to be defective as it lacks a conserved portion of *Upf2* coding sequence) tandemly into the *Drosophila* transformation vector pCaSpeR4 [50] with a unique NotI site between the fragments to give pMM#200. pMM#200 was linearized with NotI and transformed into EL250[24A2], in which the recombination machinery had been induced. Transformants were plated onto LB plates containing carbenicillin to select for gap repair of pMM#200, which can occur by homologous recombination with the BAC and result in transfer of *Upf2* into pMM#200. The resultant plasmids were analyzed by restriction digestion, and one with the expected pattern (pMM#201) was used to establish transgenic lines on the second and third chromosomes by P-element transformation. Six lines were tested and all six rescued hemizygous male *Upf2^14J^* mutants to viability. The degree of rescue varied based on insertion site, but for the strongest lines *(P{w^+^, Upf2^+^}11A* and *24)* rescued animals appeared indistinguishable from *Upf2^+^* animals and were readily maintained as stocks of genotype *Upf2^25G^; P{w^+^, Upf2^+^}11A* or *24/+* or *Upf2^14J^; P{w^+^, Upf2^+^}11A* or *24/+*.

For *GFP* reporter constructs, coding sequence of *eGFP* (Clontech; http://www.clontech.com) was amplified by PCR using KpnI linker primers Kpn5GFP and Kpn3GFP (see Table S3 for primer sequences). The product was digested with KpnI and cloned into the KpnI site of *pUAST* to generate *pUAST-eGFP*. A PCR-based strategy was used to insert a 61-bp intron derived from gene CG3585 between nucleotides 330 and 331 of the *eGFP* coding sequence to make *eGFP+I* constructs.

The intron in the SV40 3′ UTR of *pUAST* was deleted by a PCR-based strategy to give *pUASTΔI*. To replace the SV40 3′ UTR with the *hsp70* 3′ UTR, primers XbaHsp70 and Hsp70Stu were used to amplify the *hsp70* 3′ UTR from *pGATB* [27]. The PCR product was digested with XbaI and StuI and cloned between the XbaI and StuI restriction sites of *pUAST-eGFP* (to make *pUAST.h-eGFP*) or *pUAST-eGFP+I* (to make *pUAST.h-eGFP+I*).

Constructs made using PCR were sequenced to confirm that mutations had not been introduced. Constructs were transformed into *Drosophila* using standard microinjection techniques using the Δ2–3 helper plasmid as the transposase source.

### RNA analysis and quantitation.

L3 larvae of the appropriate genotype were identified by enhancement of GFP for mutant alleles or by sexing using gonad morphology for wild-type controls. Total larval RNA was prepared using Trizol reagent (Invitrogen; http://www.invitrogen.com), and genomic DNA contamination was eliminated with DNAse (DNA-free, Ambion; http://www.ambion.com). RNA concentration was determined spectrophotometrically and normalized before reverse transcription with MuMLV reverse transcriptase (Retroscript kit, Ambion). Real-time quantitative PCR was done with a thermocycler (iCycler) and real-time PCR mix (iQ SYBR Green Supermix; Bio-Rad Laboratories; http://www.bio-rad.com). Primers used in the qRT-PCR experiments were designed to amplify 60- to 100-bp fragments within single exons; amplification of *Drosophila* genomic DNA containing a *UAS-GFP* transgene showed that primers gave a linear amplification response at concentrations ranging over four orders magnitude. Control reactions performed on RNA without reverse transcription or with primers against nontranscribed regions of genomic DNA gave negligible signals compared to experimental reactions. Experimental reactions were carried out in duplicate (except EK161155 was done once) on two RNA samples that were derived independently from the RNA used for microarray analysis. Results were normalized to results with *rp18LA* transcript, a gene that is not developmentally regulated [51].

To make *Upf2^25G^* homozygotes for amplifying *tra* transcript in females, we used *Upf2* genomic rescue construct 11A on the second chromosome. *w/Y; btl-GAL4, UAS-GFP* males were crossed to *Upf2^25G^; P{w^+^, Upf2^+^}11A*/+ females; the resultant *Upf2^25G^/Y; P{w^+^, Upf2^+^}11A/btl-GAL4, UAS-GFP* males were crossed to *Upf2^25G^/FM7i, ACT-GFP* females, and female larvae with enhanced tracheal GFP expression were collected. For control larvae, we crossed the *Upf2^25G^/Y; P{w^+^, Upf2^+^}11A/btl-GAL4, UAS-GFP* males to *y w FRT^19A^* females and collected female larvae with tracheal GFP expression. cDNA from these larvae was prepared as above.

For analysis of *Adh* RNA levels, cDNA was prepared from adult males of the appropriate genotype. *Adh* transcripts were amplified with primer AdhL and the primer AdhR (for agarose gel analysis and sequencing) or AdhR2Fam (for capillary electrophoresis). PCR products were sequenced directly or digested with PvuII to distinguish the *Adh* ^+^ and *Adh^n4^* alleles. Capillary electrophoresis was performed on an ABI 3730x1 (Applied Biosystems; http://www.appliedbiosystems.com) and analyzed using GeneMapper v3.0 software (Applied Biosystems).

### Microarray analysis.

RNA was isolated from *Upf2^25G^/Y; btl-GAL4, UAS-GFP/+* and *y w FRT^19A^/Y; btl-GAL4, UAS-GFP/+* L3 larvae using Trizol as described above. cDNA labeled with Cy3 or Cy5 was prepared from each RNA sample and hybridized to microarrays containing ~14,000 gene probes [52,53]. Hybridizations were performed with two independently isolated and labeled RNA samples. Analysis was carried out using the Stanford Microarray Database (http://genome-www5.stanford.edu). During analysis we noted that the *Upf2^25G^* mutants were delayed in development. To avoid confounding effects of changes in gene expression that result from developmental regulation rather than more direct effects of *Upf2* loss of function, we used the available wild-type developmental gene expression time course [51] to filter out genes whose transcription changed more than 25% from their maximal value during hours 72–96 of larval development. The wild-type dataset includes ~33% of genes, and we used just this subset for our analysis. Furthermore, the wild-type dataset is for mixed sex populations, while our microarray was performed only on males. To compensate for sex differences, we also excluded from analysis genes that differed between males and females by more than 50% based on data from male and female larvae (E. Johnson and M. A. K., unpublished data). Our analysis of genes regulated by NMD is therefore conservative, covering only non-developmentally regulated and non-sex-regulated genes whose expression was affected by a hypomorphic *Upf2* allele, and thus provides only a lower estimate of genes regulated by NMD.

## Supporting Information

Table S1 Genes Upregulated More than 2-Fold in Microarray Analysis of *Upf2^25G^* Larval RNA (22 KB DOC)

Table S2 Genes Downregulated More than 2-Fold in Microarray Analysis of *Upf2^25G^* Larval RNA (36 KB DOC)

Table S3 Sequences of DNA Primers Used (31 KB DOC)

### Accession Numbers

The FlyBase (http://flybase.net)/Entrez Gene (http://www.ncbi.nlm.nih.gov/entrez/query.fcgi?db=gene) accession numbers for the genes described in the text are *Adh* (CG3481/Gene I.D. 3771877), *oda* (CG16747), *Smg1* (CG32743/Gene I.D. 31625), *Upf1* (CG1559/Gene I.D. 32153), and *Upf2* (CG2253/Gene I.D. 31724). Microarray data have been deposited in the National Center for Biotechnology Information Gene Expression Omnibus (http://www.ncbi.nlm.nih.gov/geo) and are accessible through accession number GSE5585.

## Abbreviations

EJC: exon junction complex
FLP: Saccharomyces cerevisiae FLP1 recombinase
FRT: FLP1 recombinase target
GFP: green fluorescent protein
L3: third larval instar
NMD: nonsense-mediated mRNA decay
*oda*: *orthinine decarboxylase antizyme*
PTC: premature termination codon
*smg*: *suppressor with morphogenetic effect on genitalia*
SNP: single nucleotide polymorphism
*tra*: *transformer*
*Upf*: *up-frameshift suppressor*
