# Supplementary material for: Functions of the Nonsense-Mediated mRNA Decay Pathway in Drosophila Development
Source: PLoS Genet. 2006 Dec 29;2(12):e180. doi: 10.1371/journal.pgen.0020180 (PMC1756896; doi:10.1371/journal.pgen.0020180)
Supplement: Table S1 — (22 KB DOC) [file pgen.0020180.st001.doc]

Table S1. Genes upregulated more than 2-fold in microarray analysis of *Upf2^25G^* larval RNA

| CG number | Gene name | Gene function or homology | Fold increase ± 1 S.D. |
| --- | --- | --- | --- |
| CG4120 | Cyp12c1 | Cytochrome P450 | 5.5±0.5 |
| CG18522 |  | Oxidoreductase/electron transporter | 4.0±0.5 |
| CG4107 | Pcaf | Histone acetyltransferase | 3.6±0.1 |
| CG16724 | transformer | Sex determination | 3.6±0.2 |
| CG18279 | Immune induced 10 | Immunity | 3.2±2.1 |
| CG16747 | gut feeling/oda | Amino acid biosynthesis regulation | 3.1±0.7 |
| CG8855 |  | Concanavalin A-like | 3.0±0.3 |
| CG8256 | G-3-P dehydrogenase | Metabolic enzyme | 2.6±0.0 |
| CG7858 | Mocs1 | Molybdenum cofactor synthesis | 2.5±0.2 |
| CG3458 | Topoisomerase 3beta | Topoisomerase | 2.4±1.2 |
| CG5962 | Arrestin B | Phototransduction | 2.2±0.8 |
| CG10916 |  |  | 2.1±0.2 |
| CG3524 | v(2)k05816 | Fatty acid biosynthesis | 2.1±0.1 |
| CG4784 |  | Cuticle component | 2.0±0.0 |
