# Supplementary material for: Functions of the Nonsense-Mediated mRNA Decay Pathway in Drosophila Development
Source: PLoS Genet. 2006 Dec 29;2(12):e180. doi: 10.1371/journal.pgen.0020180 (PMC1756896; doi:10.1371/journal.pgen.0020180)
Supplement: Table S2 — (36 KB DOC) [file pgen.0020180.st002.doc]

Table S2. Genes downregulated more than 2-fold in microarray analysis of *Upf2^25G^* larval RNA

| CG number | Gene name | Gene function or homology | Fold decrease ± 1 S.D. |
| --- | --- | --- | --- |
| CG13164 | Syntaxin Interacting Protein 2 |  | 15.0±1.7 |
| CG10859 | *jagunal* | Microtubule-based movement | 6.0±3.8 |
| CG1979 |  |  | 5.3±2.4 |
| CG10978 |  |  | 4.8±0.2 |
| CG12184 |  |  | 3.4±0.7 |
| CG11806 |  | Amino acid permease | 3.2±1.2 |
| CG12123 |  |  | 3.2±1.6 |
| CG10881 |  | Protein synthesis initiation | 3.0±0.9 |
| CG10750 |  |  | 3.0±0.9 |
| CG12035 |  |  | 2.7±1.1 |
| CG5456 | *GUK-holder* | Dlg interactor | 2.6±1.5 |
| CG11591 |  |  | 2.5±0.4 |
| CG9411 |  |  | 2.4±0.7 |
| CG1070 | *Alhambra* | Molting | 2.3±1.0 |
| CG17148 | Esterase P |  | 2.3±0.8 |
| CG7966 |  | cytochrome cd1-nitrite reductase | 2.3±0.2 |
| CG4068 |  |  | 2.2±0.1 |
| CG18132 |  | Thioredoxin-like | 2.2±0.3 |
| CG10160 | Ecdysone-inducible gene L3 |  | 2.2±0.6 |
| CG16772 |  |  | 2.2±0.9 |
| CG1322 | Zn finger homeodomain 1 | Transcription factor | 2.2±1.5 |
| CG3245 | Protein phosphatase N | Protein phosphatase | 2.2±0.3 |
| CG8483 |  | Defense response | 2.1±0.5 |
| CG3610 |  |  | 2.1±0.1 |
| CG6577 | *cannonball* | Transcription initiation | 2.1±0.1 |
| CG6255 | succinate-CoA ligase |  | 2.1±0.2 |
| CG8084 | *anachronism* | Neuroblast proliferation | 2.0±0.2 |
