# Supplementary material for: Functions of the Nonsense-Mediated mRNA Decay Pathway in Drosophila Development
Source: PLoS Genet. 2006 Dec 29;2(12):e180. doi: 10.1371/journal.pgen.0020180 (PMC1756896; doi:10.1371/journal.pgen.0020180)
Supplement: Table S3 — (31 KB DOC) [file pgen.0020180.st003.doc]

**Table S3. Sequences of DNA primers used**

# Cloning primers

Kpn5GFP 5’ GAGGTACCATGGTGAGCAAGGGCGAG

Kpn3GFP 5’ GAGGTACCCTTGTACAGCTCGTCC

XbaHsp70 5’ TCTAGAGGATCTTTGTGATAAAGCCAAATAGAAATTATTC

Hsp70Stu 5’ TAGGCCTAAACGAGTTTTTAAG

Primer pairs used for quantitative RT-PCR experiments

GFP 5’ GACAACCATTACCTGTCCACACA

5’ GGTCTCTCTTTTCGTTGGGATCT

*rp49* 5’ TCTTGTAACGTGGTCGGAATACA

5’ AATGACAATTGAACTCGGCACTC

*rp18LA* 5’ CCCACTCCATCCAGATCATTAAG

5’ GATCTTTGAATCGTGGAACTGCT

*oda* 5’ GCCACAGAAACGAAACAAAGAAG

5’ TTGCGGAAACTACGAAACAGAAT

*tra* 5’ ATACCAAAGGCTACCACGTCCTC

5’ GTAGCCAAATCGCGGAACTC

EK161155 5’ TAGTCCTGAGTTGCTCCCTATCG

5’ CAACAGGATGTGTCCAGTGATGT

*tra* primer pair

5’ TGAAAATGGATGCCGACAG

5’ CTCTTTGGCGCAATCTTCTC

*Adh* primers

AdhL 5’ GTCTGGACACCAGCAAGGAG

AdhR 5’ GGTTCAGCTCGATAGCCTTG

AdhR2Fam 5’ Fam-TGTTGACCAGGCCAGTGTAG
